# R-Methylation in Plants: A Key Regulator of Plant Development and Response to the Environment

**DOI:** 10.3390/ijms25189937

**Published:** 2024-09-14

**Authors:** Clément Barré-Villeneuve, Jacinthe Azevedo-Favory

**Affiliations:** 1 Crop Biotechnics, Department of Biosystems, KU Leuven, 3000 Leuven, Belgium; 2 KU Leuven Plant Institute (LPI), KU Leuven, 3000 Leuven, Belgium; 3 CNRS, Laboratoire Génome et Développement des Plantes, UMR 5096, 66860 Perpignan, France; 4 Laboratoire Génome et Développement des Plantes, Université Perpignan Via Domitia, UMR 5096, 66860 Perpignan, France

**Keywords:** PTM, arginine methylation, PRMT, Tudor, demethylation

## Abstract

Although arginine methylation (R-methylation) is one of the most important post-translational modifications (PTMs) conserved in eukaryotes, it has not been studied to the same extent as phosphorylation and ubiquitylation. Technical constraints, which are in the process of being resolved, may partly explain this lack of success. Our knowledge of R-methylation has recently evolved considerably, particularly in metazoans, where misregulation of the enzymes that deposit this PTM is implicated in several diseases and cancers. Indeed, the roles of R-methylation have been highlighted through the analyses of the main actors of this pathway: the PRMT writer enzymes, the TUDOR reader proteins, and potential “eraser” enzymes. In contrast, R-methylation has been much less studied in plants. Even so, it has been shown that R-methylation in plants, as in animals, regulates housekeeping processes such as transcription, RNA silencing, splicing, ribosome biogenesis, and DNA damage. R-methylation has recently been highlighted in the regulation of membrane-free organelles in animals, but this role has not yet been demonstrated in plants. The identified R-met targets modulate key biological processes such as flowering, shoot and root development, and responses to abiotic and biotic stresses. Finally, arginine demethylases activity has mostly been identified in vitro, so further studies are needed to unravel the mechanism of arginine demethylation.

## 1. Introduction

**Figure 1:**
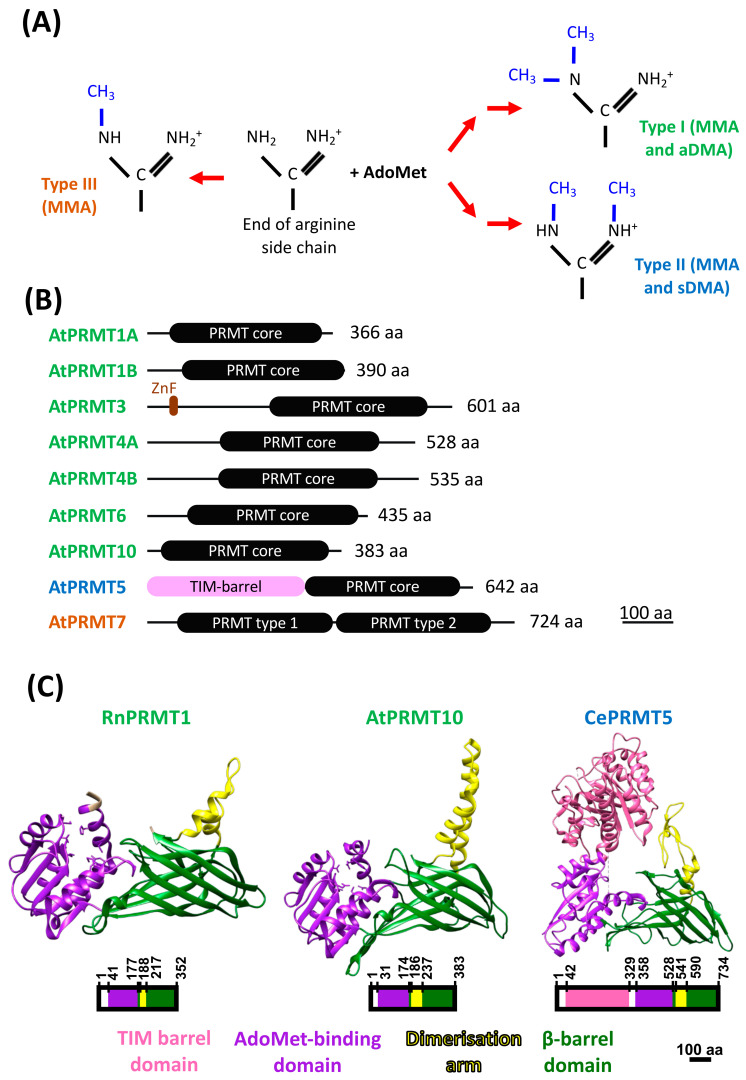
The different types of PRMTs and their structure. (**A**) Depiction of the different types of arginine methylations. MMA: monomethylation; aDMA: asymmetric dimethylation; sDMA: symmetric dimethylation. The production of dimethyl-R marks is a two-step process with an R-monomethylated intermediate form, illustrated here by the double red arrows [6]. (**B**) Linear representation of the different PRMTs of *Arabidopsis thaliana*. aa: amino acids, ZnF: zinc finger domain. Based on PROSITE database (https://prosite.expasy.org/, accessed on 31 May 2023). (**C**) The PRMT core is conserved in different PRMTs from different eukaryotes. Three-dimensional structures and linear depictions, from left to right: PRMT1 from *Rattus norvegicus*; PRMT10 from *A. thaliana*; PRMT5 from *Caenorhabditis elegans*. The AdoMet-binding domain (purple) interacts with the methyl group donor molecule (AdoMet), and the active site of PRMT catalyses the transfer of the methyl group from AdoMet to the target arginine residue of the substrate [4,17]. The active site is in a hairpin loop located between the AdoMet-binding domain and the β-barrel domain (in green) [4]. The active-site-containing structure is generally called the “double-E loop” since the key residues of this catalytic domain are two glutamate (E) residues, which are highly conserved among PRMTs [4,16]. Finally, the last domain of the PRMT core, the dimerisation arm (in yellow), allows PRMT dimerisation, which is essential for PRMT activity [4,15,17]. The TIM-barrel domain (in pink) is only present in PRMT5 homologs. The linear depictions are scaled and the residue numbers bordering each domain are labelled. The 3D structures showed come from Zhang et al. (2003) [38], Cheng et al. (2011) [17], and Sun et al. (2011) [16].

Living organisms must be able to quickly adapt their metabolism to cope with a constantly changing environment. In this context, post-translational modifications (PTMs) are an ideal way to rapidly respond to environmental signals by directly modulating protein activity, subcellular localisation, stability, and/or interactions with other molecules such as nucleic acids, lipids, or other proteins [1]. Interestingly, a type of PTM called the arginine/R-methylation has been shown to be involved in the regulation of key mechanisms, such as transcriptional regulation, splicing, RNA silencing, and ribosome biogenesis, in animals, yeast, and plants for several years [2,3,4]. R-methylation is known to be as common as phosphorylation or ubiquitylation in human cells [5]. This PTM corresponds to the addition of one or two methyl groups on the nitrogen atoms of the arginine side chain [6]. There are three main types of R-methylation marks, depending on the number of methyl groups added and the way these methyl groups are deposited to either produce monomethylated arginine (MMA), or symmetrically dimethylated arginine (sDMA) and asymmetrically dimethylated arginine (aDMA) (Figure 1A) [6].

R-methylation deposition is carried out by an enzyme family widely conserved in eukaryotes and called the PROTEIN ARGININE METHYLTRANSFERASE (PRMT) [2,3,7,8,9,10,11,12]. Indeed, PRMT homologues can be found in yeast (*Saccharomyces cerevisiae*), in animals (*Homo sapiens* and *Drosophila melanogaster*), and in plants, both in eudicots (*Arabidopsis thaliana* and *Eucalyptus grandis*) and monocots (*Oryza sativa* and *Zea mays*) [2,3,7,8,9,10,11,12,13]. The PRMTs transfer a methyl group from their co-factor, the S-adenosyl L-methionine (AdoMet) molecule, to arginine residues of protein substrates [6]. These enzymes are divided into three types: type III enzymes can only produce monomethylated arginines; type II can monomethylate and dimethylate arginine symmetrically; and type I can monomethylate and dimethylate arginine asymmetrically (Figure 1A) [6]. The *A. thaliana* genome encodes nine PRMTs, including one type III (PRMT7), one type II (PRMT5), and seven type I PRMTs (PRMT1a, PRMT1b, PRMT3, PRMT4a, PRMT4b, PRMT6, and PRMT10) (Figure 1B). Among all these PRMTs, PRMT10 is the only plant-specific PRMT [14]. The PRMT enzymes can vary significantly in length, but they all share a conserved structure, called the PRMT core (Figure 1B) [4,15]. This PRMT core contains approximately 310 amino acids and is organised into three main domains: an N-terminal AdoMet-binding domain; a dimerisation arm; and a C-terminal β-barrel domain (Figure 1C) [4,15,16,17]. In animals, most type I PRMTs and PRMT5 methylate arginine residues are located in arginine- and glycine-rich motifs known as RGG/GRG motifs, with the exception of PRMT4, which methylates arginine embedded in PGM-rich motifs (proline, glycine, and methionine) [6,18].

Methylation is a neutral and small modification that increases arginine steric hindrance and overall hydrophobicity, and thus may affect the interaction of the R-methylated protein with other proteins or nucleic acids [19]. As it does not change the global charge of the arginine residue, the R-methylated peptides are difficult to enrich, and this may explain the difficulties encountered when studying R-methylation processes [20]. In addition, it is also difficult to distinguish the two types of R-dimethylation by mass spectrometry as sDMA and aDMA are isobaric. However, an article published by Hart et al. in 2019 showed that two methods (immunoaffinity enrichment and strong cation exchange at high pH) can be used in parallel to efficiently enrich R-methylated substrates for mass spectrometry analysis [21]. The first method relies on antibodies that specifically recognise the different types of R-methylation, while the second one allows the capture of highly positively charged methyl peptides resulting from the absence of cleavage of the methylated arginine by trypsin [21]. Combining both methods is needed to obtain a correct overview of the arginine methylome [21]. In 2020, Hart et al. showed that it was possible to distinguish peptides containing sDMA and aDMA by adapting several mass spectrometry parameters [22]. These developments will contribute to increasing our knowledge of R-methylation, particularly in plants [11,23,24].

Once deposited on the target protein, R-methylation marks have been shown to be mainly recognised by members of the TUDOR domain-containing (TDRD) protein family [25,26]. In humans, 12 TDRD proteins have been identified: TDRD1, TDRD2 (also called TDRKH), TDRD4 (also called RNF17), TDRD5, TDRD6, TDRD7, TDRD8 (also called STK31), TDRD9, TDRD10, TDRD11 (also called SND1, TSN, Tudor-SN or p100), TDRD12 (also called ECAT8), and TDRD15. In plants, all the TDRD proteins identified are orthologous to SND1 [27,28,29,30,31]. The recognition of R-methylation by TDRD proteins mostly triggers changes in the subcellular localisation of proteins and regulates biological condensates in animals [32,33]. In plants, TUDOR proteins are also involved in the regulation of cytosolic condensates such as stress granules and p-bodies. Although TSN proteins have been shown to interact with R-dimethylated ARGONAUTE (AGO) proteins in a PRMT5-dependent manner in *A. thaliana* [34,35], their role as a *bona fide* reader module of R-methyl marks remains to be investigated in plants [36,37].

Finally, R-methylation has been hypothesised to be a dynamic mark, but for now, the erasing enzymes that may remove R-methylation have still not been clearly identified in vivo and the existence of specific R-demethylases is still debated [39,40,41].

In this review, we will introduce the R-meth-dependent pathways identified in plants and highlight recent developments in TDRD-type R-met readers and potential R-met erasers. We will also underline the importance of a better understanding of the R-methylation pathway in plants.

## 2. Regulation of Plant Metabolism by PRMT Proteins

### 2.1. Histone R-Methylation and Transcription Regulation Impacts on Plants

**Figure 2:**
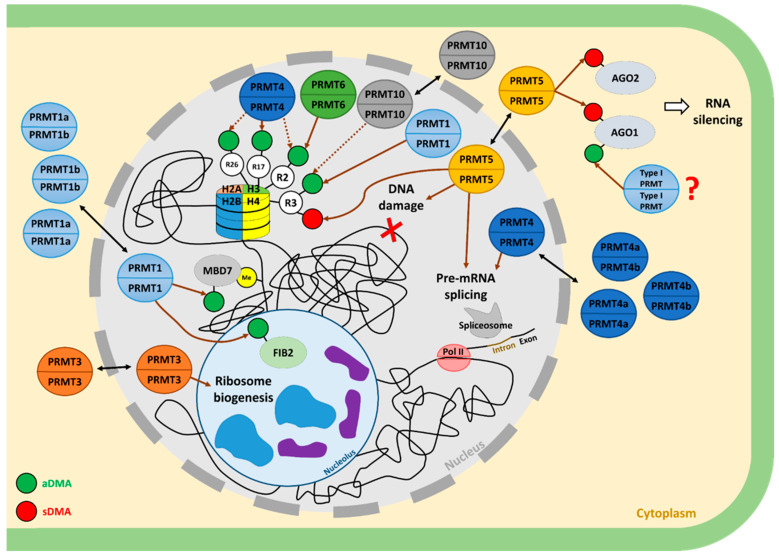
Overview of the roles of PRMTs in housekeeping mechanisms in the cell of *A. thaliana*. PRMT enzymes have been shown to be active as homodimers [17,38]. Interestingly, in *A. thaliana*, PRMT4 and PRMT1, which are duplicated as PRMT1a and PRMT1b, and PRMT4a and PRMT4b, respectively, form heterodimers [10,44]. All the PRMTs are localised in both the nucleus and cytoplasm, except for PRMT6, which is only present in the nucleus [7,10,23,43,44,51]. All the PRMTs studied in *A. thaliana*, except PRMT3, can methylate arginine residues on histone. In this context, an arginine residue can be targeted by several type I enzymes, with the exception of the H4R3 residue, which can also be modified by the PRMT5 type II enzyme. Thus, H4R3 can be regulated by PRMT1, PRMT5, and PRMT10 [10,11,14]. Histone H3 can be regulated by PRMT4 (H3R2, H3R17, and H3R26) and PRMT6 (H3R2) [43,44]. Considering non-histone targets, PRMT1 can regulate the epigenetic regulator MBD7 [52] and the nucleolar enzyme FIB2 involved in rRNA maturation [10]. PRMT3 is involved in the production of functional ribosomes [23]. Along with histone regulation, the control of mRNA splicing is the other main focus of PRMT regulation and so far involves PRMT4 and PRMT5 [53,54]. With regard to the other post-transcriptional steps, PRMT5 has also been implicated in the regulation of RNA silencing through the symmetric dimethylation of AGO1 and AGO2 [34,35]. Interestingly, AGO1 can also be asymmetrically dimethylated by one or more unknown type I PRMT(s) [35]. Finally, PRMT5 may also be involved in DNA damage response [55]. The brown dashed arrows indicate a methylation shown only in vitro. The full brown arrows indicate an involvement. The double black arrows indicate a dual localisation in the cytoplasm and in the nucleus. The red and green circles indicate symmetric (sDMA) and asymmetric dimethylation on arginine (aDMA), respectively. The yellow circle indicates DNA methylation.

In animals, several PRMTs (like PRMT1, PRMT4, PRMT5, PRMT6, and their orthologues) have been shown to methylate histone proteins such as H4, H3, and H2A [42]. Depending on the PRMT involved and the nature of the targeted locus, R-methylation can have a dual functional outcome leading either to repression or activation of transcription [6]. Interestingly, similar histone-based transcriptional regulation is conserved in plants (see details in Figure 2) [10,11,14,43,44]. Among all PRMTs, PRMT5, which is the most studied in plants, has been shown to methylate H4R3 and thus inhibits transcription [11,45]. The targeted genes are involved in the regulation of several crucial biological processes of plant development but also in plant environmental responses [11,45,46,47,48,49].

#### 2.1.1. Transcriptional Control of Plant Development by PRMT5

*FLOWERING LOCUS C* (*FLC*) gene, encoding a master regulator of flowering, is the first locus that has been identified to be regulated by H4R3 methylation [11]. Indeed, Wang et al. (2007) showed that *FLC* is upregulated in *prmt5* mutants and linked this to the decrease in sDMA on H4R3 in the promoter region of *FLC* [11]. This result may partly explain the delayed flowering time observed in *prmt5* mutants [11,12,50]. In addition, PRMT5 has also been involved in the maintenance of the shoot apical meristem (SAM) [46]. Indeed, Yue et al. (2013) showed that PRMT5 represses the expression of *CORYNE* (*CRN*) through H4R3 methylation [46]. CRN is a receptor-like kinase that transmits CLAVATA3 (CLV3) signals and is therefore involved in the *WUSCHEL* (*WUS*)*-CLV3* regulatory loop of SAM [46]. Thus, in *prmt5* mutants, *WUS* and *CLV3* are downregulated, and the SAM is smaller than in WT plants. The mutation of *CRN* rescues *WUS* expression and causes SAM size defects in *prmt5* mutant [46]. Shoot regeneration was also shown to be regulated by PRMT5, further illustrating the impact of R-methylation on shoot development [47]. In that case, Liu et al. (2016) demonstrated that PRMT5-dependent R-methylation of H4R3 leads to the repression of *KIP-RELATED PROTEIN* (*KRPs*) genes that encode a family of cell cycle factors repressing shoot regeneration [45].

#### 2.1.2. Transcriptional Control of Plant Response to Environment by PRMT5

In addition to plant development, methylation of H4R3 by PRMT5 has also been shown to be involved in plant response to the environment [45,48,49]. The data have first highlighted a contribution to the regulation of salt stress response with several salt stress-related genes being upregulated in prmt5 mutants in a non-stressed situation [48]. Among them, at least one gene, HOMOLOGY TO ABI1 (HAB1), which is known to be important for salt stress resistance, is regulated by a PRMT5-dependent deposition of a methylation mark on H4R3 [48]. PRMT5 has also been shown to regulate CAS gene transcription, which encodes a putative Ca^2+^ binding protein, which could play a role in stomatal closure and drought tolerance [45]. Indeed, Fu et al. (2013) suggested that PRMT5 binding to CAS promoters is regulated by the concentration of extracellular calcium. Indeed, increased extracellular calcium inhibits the binding of PRMT5 to the CAS promoter, which increases CAS expression and promotes stomatal closure and drought tolerance. Another example of environmental response regulated through H4R3 methylation by PRMT5 in *A. thaliana* is iron homeostasis [49]. Fan et al. (2014) demonstrated that PRMT5 can symmetrically dimethylate histone H4R3 on the Ib subgroup of bHLH genes, known to encode transcription factors that regulate iron homeostasis [49]. In case of iron deficiency, PRMT5 associates less with the chromatin of the bHLH genes, leading to an increase in their transcription [49]. In addition, the regulation of transcription by PRMT5 via H4R3 is also involved in the responses to biotic stresses, as Drozda et al. (2022) have highlighted that PRMT5 gene expression in *Solanum tuberosum* is regulated by *Phytophthora infestans* infection [56]. In this context, it has been shown that PRMT5 dynamically deposits the H4R3 mark in the promoter region of defence-related genes [56]. Finally, this work also suggested that PRMT5 plays an important role in properly regulating hypersensitive responses during *P. infestans* infection [56].

#### 2.1.3. Transcriptional Control through Histone R-Methylation by Type I PRMTs

Besides PRMT5, other PRMT enzymes are known to methylate histones and regulate transcription in animals [42]. However, although some type I enzymes have been shown to methylate histones in vitro, in plants, their role in regulating transcription in vivo is still poorly described (Figure 2) [10,14,43,44,52]. In animals, PRMT1 is the main type I PRMT which asymmetrically dimethylates H4R3 (H4R3me2s) [57,58]. In *A. thaliana*, PRMT1 orthologous enzymes, PRMT1a (or PRMT12) and PRMT1b (or PRMT11), can also produce H4R3me2s in vitro and in vivo [10,52]. But unlike animal PRMT1, PRMT1b can asymmetrically dimethylate H3 and H2A in vitro but to a lesser extent than H4 [10,52]. Although the regulation of transcription by PRMT1 via histone methylation has not yet been demonstrated in plants, it has been suggested that PRMT1b regulates transcription through methylation of METHYL-DNA-BINDING7 (MBD7), a protein involved in DNA methylation response (Figure 2) [52]. Like *PRMT1*, *PRMT4,* also known as *CARM1,* is present in *A. thaliana* through two paralogous genes: *PRMT4a* and *PRMT4b* [44]. These two proteins can asymmetrically dimethylate H3R2, H3R17, and H3R26 in vitro [44]. In *A. thaliana*, the *atprmt4a atprmt4b* double mutant shows a depletion of H3R17me2a mark, which is not observed in single mutants [44]. This may imply that H3R17me2a can be redundantly deposited by PRMT4a and PRMT4b in vivo [44]. However, the H3R17me2a mark can still be detected in the double mutant, suggesting that other PRMTs are able to produce H3R17me2a in the absence of PRMT4a and PRMT4b [44]. Indeed, Niu et al. (2008) showed that the production of H3R17me2a in vitro increases in presence of PRMT1b or PRMT10 [44]. In addition, Niu et al. (2007) have shown that PRMT10 can also asymmetrically dimethylate H4R3 in *A. thaliana*. Finally, PRMT6 has also been shown by Zhang et al. (2021) to R-methylate histones in plants [43] by depositing H3R2me2s at the promoter region of *FLOWERING LOCUS T* (*FT*), thereby promoting its expression [43].

### 2.2. Regulation of Splicing by PRMT Enzymes

PRMT enzymes have been implicated in the regulation of mRNA splicing in animals, and this role is also conserved in plants (Figure 2) [48,54,59,60,61]. Indeed, PRMT5 methylates factors that are essential for proper pre-mRNA splicing in *A. thaliana*, such as U-snRNPs (uridine-rich small nuclear RiboNucleoProtein particles), AtSmD1, D3, and AtLSM4 [48,54,59]. The U-snRNP Sm proteins are part of subcomplexes called U-snRNPs, and these U-snRNPs associate together and with the NineTeen complex to form an active spliceosome responsible for mRNA splicing [62,63]. Deng et al. (2016) showed that PRMT5 symmetrical dimethylation of snRNP Sm proteins is crucial for the recruitment of the NineTeen complex and the initiation of spliceosome activation [59]. As a consequence, the *prmt5* mutation triggers splicing defects in hundreds of genes involved in various biological processes [48,54]. Interestingly, most of the intron retention events identified in *prmt5* mutants were shown to correspond to post-transcriptionally spliced introns [64]. Therefore, PRMT5 seems to be mostly involved in post-transcriptional splicing rather than co-transcriptional splicing [64].

#### 2.2.1. Impacts of Splicing Regulation by PRMT5 on Plant Development

*PRMT5* exhibits an expression profile that oscillates throughout the day and is even conserved in plants grown in an environment where light and temperature parameters do not change, indicating that *PRMT5* is under the control of the circadian clock [51,65]. Interestingly, *prmt5* mutants exhibit lengthened periods in several circadian rhythms, including cotyledon movement and the expression of multiple clock genes and clock-controlled output genes [51,65]. Sanchez et al. (2010) showed that these defects in the circadian clock in *prmt5* mutants are, at least in part, caused by a strong alteration in alternative splicing of the core-clock gene *PSEUDO RESPONSE REGULATOR 9* (*PRR9*) [65]. Recently, PRMT5 was also shown to act together with CONSTITUTIVE PHOTOMORPHOGENIC1 (COP1), a key regulator of photomorphogenesis, to regulate post-transcriptional splicing induced by light [66]. In addition, the splicing regulation by PRMT5 is also involved in flowering. Indeed, the splicing of *FLOWERING LOCUS KH DOMAIN* (*FLK*) transcript, encoding a regulator of *FLC* gene and flowering time, is impacted by PRMT5 activity [47]. *FLK* mis-splicing induces *FLC* upregulation and promotes late flowering in *prmt5* mutants [47]. Shoot regeneration is also influenced by pre-mRNA splicing regulation by PRMT5, as shown by the impact of *prmt5* mutation on the pre-mRNA splicing of *RELATED TO KPC1* (*RPK*) genes [47]. Indeed, RPK is an E3 ubiquitin ligase that contributes to the degradation of cell cycle inhibitors, the KRP proteins, thereby repressing shoot regeneration [47].

#### 2.2.2. PRMT5 Impacts Plant Responses to Stresses through Splicing Regulation

Several stress-related transcripts are impacted by splicing defects in *prmt5* mutants. Zhang et al. (2011) suggested that PRMT5 regulation of pre-mRNA splicing contributes to the salt resistance mechanism [48]. Interestingly AtLSM4, which is one key player of pre-mRNA splicing, has been shown to be methylated by PRMT5 in response to salt stress [48]. In addition, loss of AtLSM4 induces salt stress hypersensitivity, as in *prmt5* mutants, and this phenotype can be rescued in *prmt5* mutants by complementation with a *35S:PRMT5* construct [48]. Moreover, PRMT5 can itself be regulated by nitric oxide (NO), a reactive oxygen species and signalling messenger, under abiotic and biotic stresses [67]. Hu et al. (2017) pointed out that NO can induce the S-nitrosylation of Cys-125 in PRMT5, which impacts PRMT5 methyltransferase activity under stress conditions such as salt stress [67]. However, the effect of S-nitrosylation on PRMT5 activity so far seems to be mainly important for the correct splicing of stress-response-related genes such as AT1G18160 only under stress conditions [67]. The regulation of splicing by PRMT5 seems to also be implicated in the response to pathogens [68]. Indeed, PRMT5 associates with AtsmD3 and ICln to form the methylosome complex, one of the intermediate complexes produced during U-snRNP complex formation, and Huang et al. (2016) further demonstrated that this methylosome complex negatively regulates immunity against the downy mildew oomycete pathogen [68,69]. As a result, PRMT5 contributes, through its association in the methylosome, to attenuating immunity against an oomycete pathogen [68].

#### 2.2.3. Regulation of Splicing by Other PRMTs

In plants, some splicing targets regulated by PRMT5 can also be regulated by PRMT4 enzymes [53]. Hernando et al. (2015) studied the annotated alternative splicing (AS) events of genes in *prmt4a prmt4b* double mutants and they showed that 21% of these evaluated AS events showed significant alterations [53]. Among those, Hernando et al. (2015) identified that several AS events concerned genes related to various metabolic pathways: primary metabolism, response to light, response to hormones, abiotic stress such as salt stress, RNA processing/splicing, or flowering time regulation [53]. These results highlight that PRMT4a and PRMT4b are involved in the regulation of AS events in plants. It is noteworthy that PRMT4s and PRMT5 are involved in the regulation of splicing in similar metabolic pathways [47,48,53,54,65]. To further continue on these results, Hernando et al. (2015) showed that introns annotated as alternatively spliced were more impacted than others in the *prmt4a prmt4b* double mutant and *prmt5* mutant [53]. This result suggested that PRMT4a, PRMT4b, and PRMT5 have a much greater impact on alternative than constitutive splicing [53].

### 2.3. Regulation of Ribosome Biogenesis

In animals, ribosome biogenesis is regulated by PRMT3 and this role has been shown to be conserved in plants (Figure 2) [23,69,70]. Indeed, the *prmt3* mutant in *A. thaliana* shows several phenotypes which are reminiscent of the mutants for ribosome proteins such as pointed and narrow first true leaves, serrated adult leaves, and distorted leaf vascular patterns [23]. In particular, the *prmt3* mutant produces ribosomes that are refractory to several known inhibitors of translation, suggesting changes in the ribosome structure [23]. Indeed, the polyribosome profile of the *prmt3* mutant shows an imbalance between the ratios of 60S/40S, 80S/40S, and polyribosomes, which disappears by complementing *prmt3* mutant background with a functional version of *PRMT3* [23]. In addition, Hang et al. (2014) also demonstrated that PRMT3 is required for proper pre-rRNA processing in *A. thaliana*, impacting the homeostasis between different pre-rRNA processing steps [23]. The mechanism implicated in this regulation has been better understood since 2021 with the demonstration of an interaction of PRMT3 with RIBOSOMAL PROTEIN S2 (RPS2) [71]. This interaction facilitates the dynamic assembly/disassembly of the pre-ribosomal 90S/Small Subunit (SSU) processome during ribosome biogenesis and represses nucleolar stress [71]. Interestingly, in *A. thaliana*, PRMT1a and PRMT1b may also be involved in ribosome biogenesis, as both PRMT1s are able to asymmetrically dimethylate in vitro the rRNA 2′-O-methyltranferase AtFIBRILLARIN2 (Fib2), a component of the snoRNP complex involved in ribosome biogenesis (Figure 2) [10,72].

### 2.4. Regulation of RNA Silencing

Accordingly, in animals, the PRMT5 enzyme regulate small RNA silencing by methylating P ELEMENT-INDUCED WIMPY TESTIS (PIWI) proteins, which are members of the ARGONAUTE (AGO) protein family [73,74,75]. A single case pointed out the existence of a regulation of an RNA-silencing effector by another PRMT enzyme in *Toxoplasma gondii,* an apicomplexan protozoan parasite which is an obligate parasite that evolved from secondary endosymbiosis of a red algae and has retained some plant-like relics and signatures [76,77]. Indeed, in *T. gondii*, PRMT1 has been shown to methylate TgAGO, which enables its interaction with TUDOR STAPHYLOCOCCAL NUCLEASE (TSN), complementing the weak RNA cleavage activity of TgAGO [75]. The RGG/GRG consensus motifs known to be modified by PRMTs have been detected in the N-terminal region of some RNA-silencing effectors in animals but also in plants [34,35,74,77]. In 2019, Hu et al. showed that PRMT5 is also involved in the regulation of RNA silencing in plants (Figure 2) by demonstrating that PRMT5 can symmetrically dimethylate the N-terminal RGG/GRG-rich region of ARGONAUTE2 (AGO2) in *A. thaliana* [34]. This symmetric dimethylation dampens AGO2 activity by promoting both its degradation via the 26S-proteasome and the degradation of its loaded small RNA triggered by the interaction of AGO2 with TSN proteins [34]. Hu et al. also showed that *PRMT5* transcription is inhibited upon *Pseudomonas syringae* infection. In this context, the repression exerted by PRMT5 on AGO2 is released, favouring AGO2-dependent pathogen resistance mechanisms [34,78,79]. As AGO2 is one main effector of RNA silencing involved in plant immunity, this inhibition by PRMT5 is therefore suggested to prevent the activation of the AGO2-driven pathogen defence mechanism under safe conditions [34]. In 2022, Sheng et al. showed that, in rice, AGO2 was also symmetrically dimethylated by PRMT5 and that, like in *A. thaliana*, this symmetric dimethylation promotes AGO2 degradation through the proteasome [80]. However, in *O. sativa*, PRMT5 R-methylation of AGO2 is suggested to promote defence against *Magnaporthe oryzae* infection since *prmt5* null mutants are more sensitive to infection by this fungus, while *PRMT5* overexpressing plants are more resistant [80]. In addition, *PRMT5* expression is promoted during *M. oryzae* infection [80]. All these results underline that the regulation of AGO2 by PRMT5 plays an important role in plant defence against *M. oryzae* in rice and against *P. synrigae* in *A. thaliana*, although the outputs of this regulation on AGO2 activity are different [34,80]. Recently, AGO1, the key AGO protein in plants mediating post-transcriptional gene silencing, has also been shown to be symmetrically dimethylated by PRMT5 (Figure 2) [35]. Interestingly, AGO1 also contains asymmetric dimethylation, suggesting that AGO1 is modified by a type I PRMT that has not yet been identified. Interestingly, some arginine residues in its N-terminal extension can be targeted by either a type II PRMT5 or a type I PRMT, a feature previously observed only for residue H4R3 in plants (Figure 2) [35]. This would imply the implication of one or more type I PRMTs in addition to PRMT5 in the regulation of RNA silencing in plants. Finally, PRMT5 was also shown to influence the loading of a panel of small interfering RNA into AGO1 in *A. thaliana* [35,78]. Indeed, some mis-loaded siRNAs produced from loci located in the chromosome arms were shown to be phased siRNAs (phasiRNAs), indicating that PRMT5 would promote phasiRNA loading into AGO1 in *A. thaliana* flower buds [35].

### 2.5. Involvement in DNA Damage Repair Mechanisms

PRMT5 has recently been shown to also participate in the maintenance of root stem cells in response to DNA damage in *A. thaliana* (Figure 2) [55]. Indeed, root stem cells undergo DNA damage-induced cell death in the *prmt5* mutant and, interestingly, this phenotype is enhanced in *prmt5/atm* (*ataxia-telangiectasia mutated*) and *prmt5*/atr (*ATM/RAD3-related*) double mutants [55]. As ATM and ATR are phosphatidylinositol 3-kinase-related kinases (PPI3Ks), which are early sensors and mediators of the DNA damage responses, this observation suggests a possible synergistic action of PRMT5, ATM, and ATR in the DNA damage repair pathway [55]. Therefore, these results emphasise the involvement of PRMT5 in DNA damage repair mechanisms in root stem cells of *A. thaliana* [55].

### 2.6. PRMT-Dependent Regulations in Other Plant Biological Pathways

PRMT5 has been shown recently to directly R-methylate the L-CYSTEINE DESULFHYDRASE (LCD) protein, a key enzyme in endogenous H2S production [81]. Hydrogen sulphide (H_2_S) is a recently characterised gasotransmitter that regulates several important physiological processes [81]. More precisely, Cao et al. (2022) demonstrated that the methylation of LCD by PRMT5 enhances its enzymatic activity, thereby increasing the endogenous H_2_S signal, which improves plant tolerance to cadmium stress [81]. In *Eucalyptus grandis,* other PRMTs like PRMT1, PRMT3, PRMT4, and PRMT10 have been shown to be involved in the regulation of root development since downregulation of these different *PRMTs* induced shorter roots, a decrease in the development of lateral roots, and an alteration in root hair morphology in some cases [13]. To further identify the EgPRMT targets that may explain the observed phenotypes, Plett et al. (2017) looked for R-methylated proteins in overexpressing mutants for *EgPRMT1* or *EgPRMT10* and observed that EgPRMT1 may induce the R-methylation of β-tubulin, a cytoskeleton protein involved in root hair and cellular growth [13]. They also showed later that *EgPRMT* genes are significantly differentially expressed during *E. grandis–Pisolithus albus* symbiosis, and that the R-methylation pattern is modified compared to a no-symbiosis control [82]. This suggests that EgPRMTs are involved in the colonisation of *E. grandis* roots by the ectomycorrhizal (ECM) fungus, *P. albus*, and therefore in the regulation of ECM symbiosis [82]. Moreover, the overexpression in *A. thaliana* of *ZmPRMT1*, the homologous gene to *AtPRMT5* in maize, triggers an early flowering and an increased resistance to heat stress compared to WT plants, thereby suggesting that *ZmPRMT1* may affect flowering time and heat stress response in maize [9]. Finally, it has been shown recently that PRMT enzymes can directly modify viral proteins to regulate plant defence [83]. Indeed, Zhu et al. (2024) have highlighted in tomato that PRMT6 promotes plant defence against tomato bunch stunt virus (TBSV) via the R-methylation of its viral suppressor of RNA silencing, P19 [83].

## 3. TUDOR-Domain-Containing Proteins and Conservation of Cytoplasmic Foci Formation by R-Methylation Regulation in Plants

### 3.1. TUDOR Domain and TSN Protein Structure

**Figure 3:**
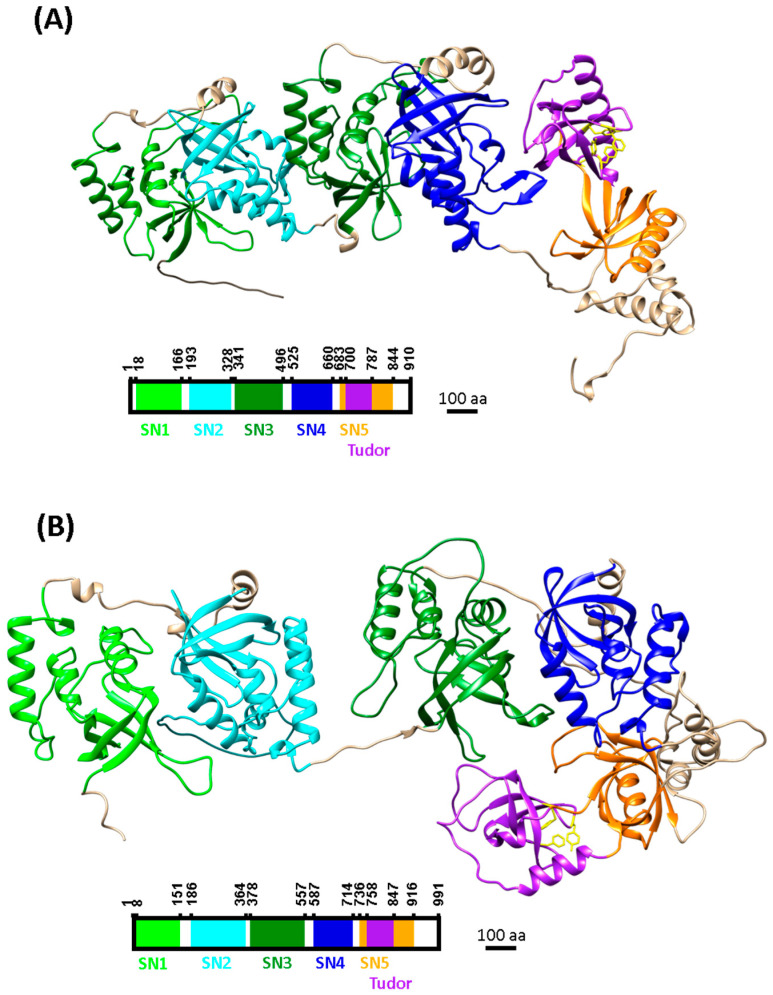
Prediction of the structures of HsSND1 and AtTSN1. AlphaFold predicted 3D structures of (**A**) SND1 from *Homo sapiens* and (**B**) TSN1 from *A. thaliana*. The different conserved domains of the HsSND1 and AtTSN1 proteins are represented by different colours; from left to right, in light green, the first SN-like domain; in cyan, the second SN-like domain; in dark green, the third SN-like domain; in dark blue, the fourth SN-like domain; and in orange, the fifth SN-like domain, which is interrupted by the canonical Tudor domain, in purple. The residues forming the aromatic cage of the canonical Tudor domain are highlighted in yellow. The positions of the residues delimiting each domain are indicated on the linear representations below. They were obtained from the UniProt database (https://www.uniprot.org/uniprotkb accessed on 31 May 2023) using Q8VZG7 TSN1_ARATH and Q7KZF4 SND1_HUMAN accessions and from information obtained in Shaw et al. (2007) [90]. The predicted 3D structures of AtTSN1 and HsSND1 are produced using AlphaFold version 2, Jumper et al. (2021) [91], and Varadi et al. (2022) [92]. The position of the residues forming the aromatic cage in HsSND1 comes from Liu et al. (2010) [84], while for AtTSN1, they were deduced from observation of the 3D structure of the canonical Tudor domain. SN: SN-like domain, aa: amino acids.

The ability of TDRD proteins to recognise R-methylated substrates is due to their extended Tudor (eTudor) domain [26,84,85,86]. This eTudor domain is composed of a canonical Tudor domain, an α-helix linker, and a staphylococcal nuclease-like domain (SN-like domain) [26]. The canonical Tudor domain (highlighted in purple in Figure 3) folds into a barrel-like structure formed by four to five β-strands containing a conserved aromatic binding pocket necessary for methyl-lysine or methyl-arginine recognition [26,86,87,88]. For TDRD11 (or SND1), this aromatic cage is mainly formed by four residues: one phenylalanine (Phe-740) and three tyrosines (Tyr-746, Tyr-763, Tyr-766) (highlighted in Figure 3) [84]. The residues forming the aromatic cage for each TDRD can vary but there are generally four and they correspond to amino acids with an aromatic ring in their side chain (phenylalanines, tyrosines, or tryptophanes) [84,85,87]. Structural studies suggest that the arginine specificity of the canonical Tudor domain in methyl-arginine readers may be due to a narrower aromatic cage than in methyl-lysine readers [88]. The other major part of the eTudor domain, the SN-like domain, displays a Tudor-like β-barrel core, but it lacks the aromatic cage [85,89]. It is interesting to note that in the case of the interaction of the eTudor domain of SND1 with sDMA, residues from the aromatic cage of the canonical Tudor domain and the SN-like domain are involved. Thus, the canonical Tudor domain and the SN-like domain of the eTudor domain are both involved in sDMA binding [84]. Finally, it has been shown that the different TDRD proteins can associate with both sDMA and aDMA, but they generally have a better affinity for one of the two types of R-dimethylation marks, for example, with sDMA for SND1 [26,84,88].

In plants, TDRD proteins have so far only been identified and characterised in *Pisum sativum*, *O. sativa*, and *A. thaliana*, and they are all orthologous of HsSND1, also called Tudor-SN [27,28,29,30,31]. In *P. sativum*, there is one HsSND1 orthologue, which is called HMP (high-molecular-weight protein) [28]. In *O. sativa*, the HsSND1 orthologue is called OsTudor-SN or Rp120 [27,29]. Finally, in *A. thaliana*, there are two HsSND1 orthologues, called TUDOR STAPHYLOCOCCAL NUCLEASE 1 (AtTSN1) and AtTSN2 [30,31]. All these plant Tudor-SN proteins are highly similar (equal to or greater than 79% of similarity) and highly identical (equal to or greater than 65% of identity) [28]. To finish, as HsSND1, AtTSN1, OsTudor-SN, and HMP possess four SN-like domains from the C-ter to N-ter ends, and in the C-ter, there is a fifth SN-like domain interrupted by a Tudor domain, the association of both domains results in the formation of an eTudor domain (Figure 3) [27,28,90,93,94]. The four SN-like domains of Tudor-SN proteins have been shown to possess RNA binding activity and nucleolytic activity, although they lack some key residues normally required for nucleolytic activity [36,95,96,97].

### 3.2. Contribution of R-Methylation through TUDOR-Domain-Containing Proteins to the Regulation of Liquid–Liquid Phase Separation

In animals, TDRD proteins’ ability to recognise R-methylation marks has been implicated in the regulation of membraneless organelle (MLO) formation [32]. Thus, Tudor domains of several TDRD proteins (HsSMN, HsSND1, HsTDRD1, HsTDRD3, HsTDRD6, and HsTDRD8) were recently shown to be involved in the formation of condensates [32]. However, they still show different behaviours. For example, when the ability of the Tudor domain in SND1 to bind sDMA is abolished, its localisation changes, or when the deposition of aDMA is inhibited, only the cytoplasmic condensates formed by the Tudor domain of TDRD3 disappear and not its nuclear condensates [32]. The R-methylation binding property of these different Tudor domains is necessary for the correct formation of condensates [32]. Among all these Tudor domains, SMN’s Tudor domain is involved in regulating the formation of Cajal bodies and gems [32]. Moreover, depending of the sDMA and aDMA level, SMN’s Tudor domain can regulate the composition of Cajal bodies and the fusion of a Cajal body and gem into a single nuclear body [32]. In addition to gems and Cajal bodies, TDRD proteins have also been shown to regulate the aggregation dynamics of stress granules in animals [33,98]. Interestingly, this global role of TDRDs in regulating cellular condensates seems to be conserved in plants [29,36,37,99]. Indeed, in *O. sativa*, OsTudor-SN has been shown to regulate the formation of specific condensates in rice seeds, called the protein body–endoplasmic reticulum (PB-ER) [29,99]. OsTudor-SN is involved in recruiting different proteins to the PB-ER and its TUDOR domain is important for this recruitment [99]. In *A. thaliana*, AtTSNs are also involved in the formation of two types of condensates: stress granules (SGs) and processing bodies (p-bodies) [36]. In addition, AtTSNs have been shown to act as scaffolding proteins for SGs, facilitating the recruitment of different proteins to SGs [37]. Although, the Tudor domain has not yet been formally implicated in this regulation of stress granules and p-bodies in *A. thaliana* [36,37], its contribution to TSN regulation of these condensates in *O. sativa* and in humans may suggest that the Tudor domain of AtTSNs may also play a role in the regulation of MLOs in *A. thaliana* [32,99].

## 4. The Hypothesis about the “Erasing” of R-Methylation

First, it was thought that R-methylation was a stable post-translational modification [2,100]. It was therefore thought that the only way to demethylate a protein was to degrade it, and then to produce a new one without any R-methylation mark [2]. However, several studies in metazoan showed that the R-methylation of different proteins can be dynamic and evolve over time [100,101,102,103,104,105]. This was the starting point for the hypothesis that arginine demethylases should exist [100]. To follow this hypothesis, Wang et al. (2004) reported PEPTIDYL ARGININE DEIMINASE 4 (PAD4) as a possible arginine demethylase [106]. PAD4 is an enzyme catalysing the hydrolytic deimination of arginine to citrulline in proteins [107]. However, the arginine demethylating activity of PAD4 was later discarded because arginine demethylation activity interferes with its citrulline conversion activity, which impairs PAD4 efficiency for arginine demethylation [108,109,110]. In 2007, Chang et al. identified another possible arginine demethylase: the 2OG oxygenase, JUMONJI-DOMAIN-CONTAINING PROTEIN 6 (JMJD6) [111]. JMJD6 is part of the enzyme superfamily of Fe(II) and 2-oxoglutarate-dependent oxygenases (2OG oxygenases), known to be composed of enzymes catalysing the hydroxylation of amino acids or demethylation of lysine residues [100]. JMJD6 was first characterised to be able to demethylate histone H3R2 and H4R3 in vitro [111]. However, this discovery led to a controversial discussion in the literature about JMJD6 enzymatic activity since it was also shown that JMJD6 can have lysine hydroxylation activity [100]. The problem was therefore to know which of these two activities was the primary and real one of JMJD6 [100]. Still, arginine demethylation of H4R3 by JMJD6 was further confirmed by Liu et al. (2013) [112], and now several other substrates of JMJD6 arginine demethylation have been identified [33,100,113,114,115,116,117]. In 2016, arginine demethylation activity was also suggested for a subset of JMJC histone lysine demethylase [40]. Walport et al. (2016) tested the capacity of truncated recombinant proteins containing the catalytic domain representative of the six identified human JMJC KDM (KDM stands for lysine demethylase) subfamilies to demethylate R/arginine on the H3 tail [40]. They used recombinant KDM2A, KDM3A, KDM4E, KDM5C, KDM6B, and PHF8 (also known as KDM7B) and modified the H3 tail, with methylated arginines (MMA, aDMA, or sDMA) instead of methylated lysines [40]. Then, the results of this test were assessed by MALDI-TOF mass spectrometry [40]. Thus, they identified that only KDM3A, KDM4E, KDM5C, and KDM6B exhibit arginine demethylation activity [40]. Then, only KDM4E and KDM5C were confirmed in their ability to demethylate arginine by another method using the same parameters as before but, this time, with a histone tail containing a known methylated arginine [40]. These first results for new arginine demethylase activity obtained in vitro were further supported by a recent analysis of Bonnici et al. (2023) [41]. Indeed, in this article, they demonstrated in vitro that all human KDM5s (KDM5A-KDM5D), KDM4E, and, to a lesser extent, KDM4A/D exhibit both lysine and arginine demethylase activities on histone peptides [41]. So, several possible arginine demethylases may have been identified other than JMJD6, but they still need to be further studied and confirmed by in vivo analyses [40,41]. Finally, in *A. thaliana*, arginine demethylation activity was also spotted for JMJ20 and JMJ22 [39]. Indeed, Cho et al. (2012) demonstrated that JMJ20 and JMJ22 can demethylate histone H4R3 in response to red light stimulus at GA3ox1/GA3ox2 chromatin, which promotes seed germination [39]. This result is still currently the only example of arginine demethylation in plants [39].

## 5. Discussion

R-methylation is involved in the regulation of fundamental mechanisms and critical biological processes of plant development and adaptation to environment [10,11,13,14,43,44,45,46,47,48,49,51,53,55,56,65,66,67,68]. To date, PRMT5 is the PRMT most implicated in the regulation of these different functions in plants. However, the lack of studies on these other PRMTs compared to PRMT5 may explain why their exact functions in plants are still poorly understood. The specificity of target recognition by PRMT5 can require a third partner in animals. These modular adaptor proteins recruit PRMT5 via a conserved PRMT5 binding motif (PBM) and bring the substrate into close proximity with PRMT5 [118,119,120,121,122,123,124]. Furthermore, in animals, the R-methylation activity of PRMT5 only occurs when it is associated with the MEP50 protein (also known as WRD77) in a tetrameric complex composed of heterodimers [16,125]. To our knowledge, such adaptor modules and cofactors have not yet been identified in plants. Therefore, understanding how PRMT enzymes recruit their targets in plants will be an important question for the future.

The activity of PRMT enzymes in animals can be regulated by phosphorylation, ubiquitination, R-methylation, or automethylation [126,127,128,129]. In plants, PRMT5 activity is regulated by S-nitrosylation, and PRMT10 activity has been suggested to be regulated by R-methylation [17,67]. However, these are the few examples of PRMT regulation in plants, and more investigations will be required to know how to modulate their activity through PTM.

Recently, in animals, R-methylation was underlined as regulating the formation of membraneless organelles thanks to the TUDOR domain of TDRD proteins [32]. Interestingly, in plants, orthologues of the TDRD protein, HsSND1, have been shown to regulate the formation of different types of condensates such as stress granules, p-bodies in *A. thaliana*, or PB-ER in *O. sativa* [29,36,37,99]. However, further studies will be required to demonstrate that recognition of R methylation by TDRD proteins is indeed involved in the formation of membrane-free organelles in plants.

Now, the research on arginine demethylation suggests that at least JMJD6 could be a promising candidate for arginine demethylase activity in animals [33,111,112,113,114,115,116]. In plants, JMJ20 and JMJ22 have previously been identified as arginine demethylases [39]. Nevertheless, further analyses are needed to better understand their biological roles and the existence of other arginine demethylases in plants.

In conclusion, this review highlights the importance of PRMTs in plants, as well as the progress that still needs to be made to better understand how R-met is set up and regulated, and to identify new targets and pathways regulated by R-met, particularly those that may be plant-specific.

## References

[1] Friso G., Van Wijk K.J. (2015). Update: Post-Translational Protein Modifications in Plant Metabolism. Plant Physiol..

[2] Bedford M.T., Clarke S.G. (2009). Protein Arginine Methylation in Mammals: Who, What, and Why. Mol. Cell.

[3] Low J.K.K., Wilkins M.R. (2012). Protein Arginine Methylation in *Saccharomyces cerevisiae*. FEBS J..

[4] Ahmad A., Cao X. (2012). Plant PRMTs Broaden the Scope of Arginine Methylation. J. Genet. Genom..

[5] Larsen S.C., Sylvestersen K.B., Mund A., Lyon D., Mullari M., Madsen M.V., Daniel J.A., Jensen L.J., Nielsen M.L. (2016). Proteome-Wide Analysis of Arginine Monomethylation Reveals Widespread Occurrence in Human Cells. Sci. Signal..

[6] Blanc R.S., Richard S. (2017). Arginine Methylation: The Coming of Age. Mol. Cell.

[7] Ahmad A., Dong Y., Cao X. (2011). Characterization of the PRMT Gene Family in Rice Reveals Conservation of Arginine Methylation. PLoS ONE.

[8] Wang Y., Li C. (2012). Evolutionarily Conserved Protein Arginine Methyltransferases in Non-mammalian Animal Systems. FEBS J..

[9] Ling Q., Liao J., Liu X., Zhou Y., Qian Y. (2022). Genome-Wide Identification of Maize Protein Arginine Methyltransferase Genes and Functional Analysis of ZmPRMT1 Reveal Essential Roles in Arabidopsis Flowering Regulation and Abiotic Stress Tolerance. Int. J. Mol. Sci..

[10] Yan D., Zhang Y., Niu L., Yuan Y., Cao X. (2007). Identification and Characterization of Two Closely Related Histone H4 Arginine 3 Methyltransferases in *Arabidopsis thaliana*. Biochem. J..

[11] Wang X., Zhang Y., Ma Q., Zhang Z., Xue Y., Bao S., Chong K. (2007). SKB1-Mediated Symmetric Dimethylation of Histone H4R3 Controls Flowering Time in Arabidopsis. EMBO J..

[12] Pei Y., Niu L., Lu F., Liu C., Zhai J., Kong X., Cao X. (2007). Mutations in the Type II Protein Arginine Methyltransferase AtPRMT5 Result in Pleiotropic Developmental Defects in Arabidopsis. Plant Physiol..

[13] Plett K.L., Raposo A.E., Bullivant S., Anderson I.C., Piller S.C., Plett J.M. (2017). Root Morphogenic Pathways in Eucalyptus Grandis Are Modified by the Activity of Protein Arginine Methyltransferases. BMC Plant Biol..

[14] Niu L., Lu F., Pei Y., Liu C., Cao X. (2007). Regulation of Flowering Time by the Protein Arginine Methyltransferase AtPRMT10. EMBO Rep..

[15] Cheng X., Collins R.E., Zhang X. (2005). Structural and Sequence Motifs of Protein (Histone) Methylation Enzymes. Annu. Rev. Biophys. Biomol. Struct..

[16] Sun L., Wang M., Lv Z., Yang N., Liu Y., Bao S., Gong W., Xu R.-M. (2011). Structural Insights into Protein Arginine Symmetric Dimethylation by PRMT5. Proc. Natl. Acad. Sci. USA.

[17] Cheng Y., Frazier M., Lu F., Cao X., Redinbo M.R. (2011). Crystal Structure of the Plant Epigenetic Protein Arginine Methyltransferase 10. J. Mol. Biol..

[18] Al-Hamashi A.A., Diaz K., Huang R. (2020). Non-Histone Arginine Methylation by Protein Arginine Methyltransferases. Curr. Protein Pept. Sci..

[19] Peng C., Wong C.C. (2017). The Story of Protein Arginine Methylation: Characterization, Regulation, and Function. Expert. Rev. Proteom..

[20] Evich M., Stroeva E., Zheng Y.G., Germann M.W. (2016). Effect of Methylation on the Side-Chain p *K*
_a_ Value of Arginine: Effect of Methylation on Arginine p *K*
_a_ Values. Protein Sci..

[21] Hartel N.G., Chew B., Qin J., Xu J., Graham N.A. (2019). Deep Protein Methylation Profiling by Combined Chemical and Immunoaffinity Approaches Reveals Novel PRMT1 Targets. Mol. Cell. Proteom..

[22] Hartel N.G., Liu C.Z., Graham N.A. (2020). Improved Discrimination of Asymmetric and Symmetric Arginine Dimethylation by Optimization of the Normalized Collision Energy in LC-MS Proteomics. J. Proteome Res..

[23] Hang R., Liu C., Ahmad A., Zhang Y., Lu F., Cao X. (2014). *Arabidopsis* Protein Arginine Methyltransferase 3 Is Required for Ribosome Biogenesis by Affecting Precursor Ribosomal RNA Processing. Proc. Natl. Acad. Sci. USA.

[24] Liang Q., Geng Q., Jiang L., Liang M., Li L., Zhang C., Wang W. (2020). Protein Methylome Analysis in Arabidopsis Reveals Regulation in RNA-Related Processes. J. Proteom..

[25] Gayatri S., Bedford M.T. (2014). Readers of Histone Methylarginine Marks. Biochim. Biophys. Acta (BBA)—Gene Regul. Mech..

[26] Gan B., Chen S., Liu H., Min J., Liu K. (2019). Structure and Function of eTudor Domain Containing TDRD Proteins. Crit. Rev. Biochem. Mol. Biol..

[27] Sami-Subbu R., Choi S.-B., Wu Y., Wang C., Okita T.W. (2001). Identification of a Cytoskeleton-Associated 120 kDa RNA-Binding Protein in Developing Rice Seeds. Plant Mol. Biol..

[28] Abe S., Sakai M., Yagi K., Hagino T., Ochi K., Shibata K., Davies E. (2003). A Tudor Protein with Multiple SNc Domains from Pea Seedlings: Cellular Localization, Partial Characterization, Sequence Analysis, and Phylogenetic Relationships. J. Exp. Bot..

[29] Wang C., Washida H., Crofts A.J., Hamada S., Katsube-Tanaka T., Kim D., Choi S., Modi M., Singh S., Okita T.W. (2008). The Cytoplasmic-localized, Cytoskeletal-associated RNA Binding Protein *Os* Tudor-SN: Evidence for an Essential Role in Storage Protein RNA Transport and Localization. Plant J..

[30] Dit Frey N.F., Muller P., Jammes F., Kizis D., Leung J., Perrot-Rechenmann C., Bianchi M.W. (2010). The RNA Binding Protein Tudor-SN Is Essential for Stress Tolerance and Stabilizes Levels of Stress-Responsive mRNAs Encoding Secreted Proteins in *Arabidopsis*. Plant Cell.

[31] Liu S., Jia J., Gao Y., Zhang B., Han Y. (2010). The AtTudor2, a Protein with SN-Tudor Domains, Is Involved in Control of Seed Germination in Arabidopsis. Planta.

[32] Courchaine E.M., Barentine A.E.S., Straube K., Lee D.-R., Bewersdorf J., Neugebauer K.M. (2021). DMA-Tudor Interaction Modules Control the Specificity of in Vivo Condensates. Cell.

[33] Gao X., Fu X., Song J., Zhang Y., Cui X., Su C., Ge L., Shao J., Xin L., Saarikettu J. (2015). Poly(A) ^+^ mRNA-binding Protein Tudor-SN Regulates Stress Granules Aggregation Dynamics. FEBS J..

[34] Hu P., Zhao H., Zhu P., Xiao Y., Miao W., Wang Y., Jin H. (2019). Dual Regulation of Arabidopsis AGO2 by Arginine Methylation. Nat. Commun..

[35] Barre-Villeneuve C., Laudié M., Carpentier M.-C., Kuhn L., Lagrange T., Azevedo-Favory J. (2024). The Unique Dual Targeting of AGO1 by Two Types of PRMT Enzymes Promotes phasiRNA Loading in *Arabidopsis thaliana*. Nucleic Acids Res..

[36] Gutierrez-Beltran E., Moschou P.N., Smertenko A.P., Bozhkov P.V. (2015). Tudor Staphylococcal Nuclease Links Formation of Stress Granules and Processing Bodies with mRNA Catabolism in Arabidopsis. Plant Cell.

[37] Gutierrez-Beltran E., Elander P.H., Dalman K., Dayhoff G.W., Moschou P.N., Uversky V.N., Crespo J.L., Bozhkov P.V. (2021). Tudor Staphylococcal Nuclease Is a Docking Platform for Stress Granule Components and Is Essential for SnRK1 Activation in *Arabidopsis*. EMBO J..

[38] Zhang X., Cheng X. (2003). Structure of the Predominant Protein Arginine Methyltransferase PRMT1 and Analysis of Its Binding to Substrate Peptides. Structure.

[39] Cho J.-N., Ryu J.-Y., Jeong Y.-M., Park J., Song J.-J., Amasino R.M., Noh B., Noh Y.-S. (2012). Control of Seed Germination by Light-Induced Histone Arginine Demethylation Activity. Dev. Cell.

[40] Walport L.J., Hopkinson R.J., Chowdhury R., Schiller R., Ge W., Kawamura A., Schofield C.J. (2016). Arginine Demethylation Is Catalysed by a Subset of JmjC Histone Lysine Demethylases. Nat. Commun..

[41] Bonnici J., Oueini R., Salah E., Johansson C., Schofield C.J., Kawamura A. (2023). The Catalytic Domains of All Human KDM5 JmjC Demethylases Catalyse N-methyl Arginine.Pdf. FEBS Lett..

[42] Di Lorenzo A., Bedford M.T. (2011). Histone Arginine Methylation. FEBS Lett..

[43] Zhang P., Li X., Wang Y., Guo W., Chachar S., Riaz A., Geng Y., Gu X., Yang L. (2021). PRMT6 Physically Associates with Nuclear Factor Y to Regulate Photoperiodic Flowering in Arabidopsis. aBIOTECH.

[44] Niu L., Zhang Y., Pei Y., Liu C., Cao X. (2008). Redundant Requirement for a Pair of PROTEIN ARGININE METHYLTRANSFERASE4 Homologs for the Proper Regulation of Arabidopsis Flowering Time. Plant Physiol..

[45] Fu Y.-L., Zhang G.-B., Lv X.-F., Guan Y., Yi H.-Y., Gong J.-M. (2013). *Arabidopsis* Histone Methylase CAU1/PRMT5/SKB1 Acts as an Epigenetic Suppressor of the Calcium Signaling Gene *CAS* to Mediate Stomatal Closure in Response to Extracellular Calcium. Plant Cell.

[46] Yue M., Li Q., Zhang Y., Zhao Y., Zhang Z., Bao S. (2013). Histone H4R3 Methylation Catalyzed by SKB1/PRMT5 Is Required for Maintaining Shoot Apical Meristem. PLoS ONE.

[47] Liu H., Ma X., Han H.N., Hao Y.J., Zhang X.S. (2016). AtPRMT5 Regulates Shoot Regeneration through Mediating Histone H4R3 Dimethylation on KRPs and Pre-mRNA Splicing of RKP in Arabidopsis. Mol. Plant.

[48] Zhang Z., Zhang S., Zhang Y., Wang X., Li D., Li Q., Yue M., Li Q., Zhang Y., Xu Y. (2011). *Arabidopsis* Floral Initiator SKB1 Confers High Salt Tolerance by Regulating Transcription and Pre-mRNA Splicing through Altering Histone H4R3 and Small Nuclear Ribonucleoprotein LSM4 Methylation. Plant Cell.

[49] Fan H., Zhang Z., Wang N., Cui Y., Sun H., Liu Y., Wu H., Zheng S., Bao S., Ling H. (2014). SKB1/PRMT 5-mediated Histone H4R3 Dimethylation of Ib Subgroup bHLH Genes Negatively Regulates Iron Homeostasis in *Arabidopsis thaliana*. Plant J..

[50] Qiu C., Wang T., Wang H., Tao Z., Wang C., Ma J., Li S., Zhao Y., Liu J., Li P. (2024). SISTER OF FCA Physically Associates with SKB1 to Regulate Flowering Time in Arabidopsis Thaliana. BMC Plant Biol..

[51] Hong S., Song H.-R., Lutz K., Kerstetter R.A., Michael T.P., McClung C.R. (2010). Type II Protein Arginine Methyltransferase 5 (PRMT5) Is Required for Circadian Period Determination in *Arabidopsis thaliana*. Proc. Natl. Acad. Sci. USA.

[52] Scebba F., De Bastiani M., Bernacchia G., Andreucci A., Galli A., Pitto L. (2007). PRMT11: A New Arabidopsis MBD7 Protein Partner with Arginine Methyltransferase Activity. Plant J..

[53] Hernando C.E., Sanchez S.E., Mancini E., Yanovsky M.J. (2015). Genome Wide Comparative Analysis of the Effects of PRMT5 and PRMT4/CARM1 Arginine Methyltransferases on the Arabidopsis Thaliana Transcriptome. BMC Genom..

[54] Deng X., Gu L., Liu C., Lu T., Lu F., Lu Z., Cui P., Pei Y., Wang B., Hu S. (2010). Arginine Methylation Mediated by the *Arabidopsis* Homolog of PRMT5 Is Essential for Proper Pre-mRNA Splicing. Proc. Natl. Acad. Sci. USA.

[55] Li Q., Zhao Y., Yue M., Xue Y., Bao S. (2016). The Protein Arginine Methylase 5 (PRMT5/SKB1) Gene Is Required for the Maintenance of Root Stem Cells in Response to DNA Damage. J. Genet. Genom..

[56] Drozda A., Kurpisz B., Arasimowicz-Jelonek M., Kuźnicki D., Jagodzik P., Guan Y., Floryszak-Wieczorek J. (2022). Nitric Oxide Implication in Potato Immunity to Phytophthora Infestans via Modifications of Histone H3/H4 Methylation Patterns on Defense Genes. Int. J. Mol. Sci..

[57] Dhar S., Vemulapalli V., Patananan A.N., Huang G.L., Di Lorenzo A., Richard S., Comb M.J., Guo A., Clarke S.G., Bedford M.T. (2013). Loss of the Major Type I Arginine Methyltransferase PRMT1 Causes Substrate Scavenging by Other PRMTs. Sci. Rep..

[58] Pawlak M.R., Scherer C.A., Chen J., Roshon M.J., Ruley H.E. (2000). Arginine *N*-Methyltransferase 1 Is Required for Early Postimplantation Mouse Development, but Cells Deficient in the Enzyme Are Viable. Mol. Cell. Biol..

[59] Deng X., Lu T., Wang L., Gu L., Sun J., Kong X., Liu C., Cao X. (2016). Recruitment of the NineTeen Complex to the Activated Spliceosome Requires AtPRMT5. Proc. Natl. Acad. Sci. USA.

[60] Meister G. (2002). Assisted RNP Assembly: SMN and PRMT5 Complexes Cooperate in the Formation of Spliceosomal UsnRNPs. EMBO J..

[61] Ohkura N., Takahashi M., Yaguchi H., Nagamura Y., Tsukada T. (2005). Coactivator-Associated Arginine Methyltransferase 1, CARM1, Affects Pre-mRNA Splicing in an Isoform-Specific Manner. J. Biol. Chem..

[62] Wahl M.C., Will C.L., Lührmann R. (2009). The Spliceosome: Design Principles of a Dynamic RNP Machine. Cell.

[63] Chanarat S., Sträßer K. (2013). Splicing and beyond: The Many Faces of the Prp19 Complex. Biochim. Biophys. Acta (BBA)—Mol. Cell Res..

[64] Jia J., Long Y., Zhang H., Li Z., Liu Z., Zhao Y., Lu D., Jin X., Deng X., Xia R. (2020). Post-Transcriptional Splicing of Nascent RNA Contributes to Widespread Intron Retention in Plants. Nat. Plants.

[65] Sanchez S.E., Petrillo E., Beckwith E.J., Zhang X., Rugnone M.L., Hernando C.E., Cuevas J.C., Godoy Herz M.A., Depetris-Chauvin A., Simpson C.G. (2010). A Methyl Transferase Links the Circadian Clock to the Regulation of Alternative Splicing. Nature.

[66] Yan Y., Luo H., Qin Y., Yan T., Jia J., Hou Y., Liu Z., Zhai J., Long Y., Deng X. (2024). Light Controls Mesophyll-Specific Post-Transcriptional Splicing of Photoregulatory Genes by AtPRMT5. Proc. Natl. Acad. Sci. USA.

[67] Hu J., Yang H., Mu J., Lu T., Peng J., Deng X., Kong Z., Bao S., Cao X., Zuo J. (2017). Nitric Oxide Regulates Protein Methylation during Stress Responses in Plants. Mol. Cell.

[68] Huang S., Balgi A., Pan Y., Li M., Zhang X., Du L., Zhou M., Roberge M., Li X. (2016). Identification of Methylosome Components as Negative Regulators of Plant Immunity Using Chemical Genetics. Mol. Plant.

[69] Bachand F., Silver P.A. (2004). PRMT3 Is a Ribosomal Protein Methyltransferase That Affects the Cellular Levels of Ribosomal Subunits. EMBO J..

[70] Swiercz R., Person M.D., Bedford M.T. (2005). Ribosomal Protein S2 Is a Substrate for Mammalian PRMT3 (Protein Arginine Methyltransferase 3). Biochem. J..

[71] Hang R., Wang Z., Yang C., Luo L., Mo B., Chen X., Sun J., Liu C., Cao X. (2021). Protein Arginine Methyltransferase 3 Fine-Tunes the Assembly/Disassembly of Pre-Ribosomes to Repress Nucleolar Stress by Interacting with RPS2B in Arabidopsis. Mol. Plant.

[72] Barneche F., Steinmetz F., Echeverría M. (2000). Fibrillarin Genes Encode Both a Conserved Nucleolar Protein and a Novel Small Nucleolar RNA Involved in Ribosomal RNA Methylation inArabidopsis Thaliana. J. Biol. Chem..

[73] Nishida K.M., Okada T.N., Kawamura T., Mituyama T., Kawamura Y., Inagaki S., Huang H., Chen D., Kodama T., Siomi H. (2009). Functional Involvement of Tudor and dPRMT5 in the piRNA Processing Pathway in Drosophila Germlines. EMBO J..

[74] Kirino Y., Kim N., De Planell-Saguer M., Khandros E., Chiorean S., Klein P.S., Rigoutsos I., Jongens T.A., Mourelatos Z. (2009). Arginine Methylation of Piwi Proteins Catalysed by dPRMT5 Is Required for Ago3 and Aub Stability. Nat. Cell Biol..

[75] Musiyenko A., Majumdar T., Andrews J., Adams B., Barik S. (2012). PRMT1 Methylates the Single Argonaute of Toxoplasma Gondii and Is Important for the Recruitment of Tudor Nuclease for Target RNA Cleavage by Antisense Guide RNA: Arg-Methylation of Protozoan Ago Recruits Tudor Slicer to RISC. Cell. Microbiol..

[76] Nagamune K., Xiong L., Chini E., Sibley L.D. (2008). Plants, Endosymbionts and Parasites: Abscisic Acid and Calcium Signaling. Commun. Integr. Biol..

[77] Martín-Merchán A., Lavatelli A., Engler C., González-Miguel V.M., Moro B., Rosano G.L., Bologna N.G. (2024). Arabidopsis AGO1 N-Terminal Extension Acts as an Essential Hub for PRMT5 Interaction and Post-Translational Modifications. Nucleic Acids Res..

[78] Seo J.-K., Wu J., Lii Y., Li Y., Jin H. (2013). Contribution of Small RNA Pathway Components in Plant Immunity. MPMI.

[79] Weiberg A., Wang M., Bellinger M., Jin H. (2014). Small RNAs: A New Paradigm in Plant-Microbe Interactions. Annu. Rev. Phytopathol..

[80] Sheng C., Li X., Xia S., Zhang Y., Yu Z., Tang C., Xu L., Wang Z., Zhang X., Zhou T. (2023). An OsPRMT5-OsAGO2 */* miR1875 *-OsHXK1* Module Regulates Rice Immunity to Blast Disease. J. Integr. Plant Biol..

[81] Cao H., Liang Y., Zhang L., Liu Z., Liu D., Cao X., Deng X., Jin Z., Pei Y. (2022). AtPRMT5-Mediated AtLCD Methylation Improves Cd2+ Tolerance via Increased H2S Production in Arabidopsis. Plant Physiol..

[82] Plett K.L., Raposo A.E., Anderson I.C., Piller S.C., Plett J.M. (2019). Protein Arginine Methyltransferase Expression Affects Ectomycorrhizal Symbiosis and the Regulation of Hormone Signaling Pathways. MPMI.

[83] Zhu Q., Ahmad A., Shi C., Tang Q., Liu C., Ouyang B., Deng Y., Li F., Cao X. (2024). Protein Arginine Methyltransferase 6 Mediates Antiviral Immunity in Plants. Cell Host Microbe.

[84] Liu K., Chen C., Guo Y., Lam R., Bian C., Xu C., Zhao D.Y., Jin J., MacKenzie F., Pawson T. (2010). Structural Basis for Recognition of Arginine Methylated Piwi Proteins by the Extended Tudor Domain. Proc. Natl. Acad. Sci. USA.

[85] Liu H., Wang J.-Y.S., Huang Y., Li Z., Gong W., Lehmann R., Xu R.-M. (2010). Structural Basis for Methylarginine-Dependent Recognition of Aubergine by Tudor. Genes Dev..

[86] Sprangers R., Groves M.R., Sinning I., Sattler M. (2003). High-Resolution X-Ray and NMR Structures of the SMN Tudor Domain: Conformational Variation in the Binding Site for Symmetrically Dimethylated Arginine Residues. J. Mol. Biol..

[87] Tripsianes K., Madl T., Machyna M., Fessas D., Englbrecht C., Fischer U., Neugebauer K.M., Sattler M. (2011). Structural Basis for Dimethylarginine Recognition by the Tudor Domains of Human SMN and SPF30 Proteins. Nat. Struct. Mol. Biol..

[88] Liu K., Guo Y., Liu H., Bian C., Lam R., Liu Y., Mackenzie F., Rojas L.A., Reinberg D., Bedford M.T. (2012). Crystal Structure of TDRD3 and Methyl-Arginine Binding Characterization of TDRD3, SMN and SPF30. PLoS ONE.

[89] Zhang H., Liu K., Izumi N., Huang H., Ding D., Ni Z., Sidhu S.S., Chen C., Tomari Y., Min J. (2017). Structural Basis for Arginine Methylation-Independent Recognition of PIWIL1 by TDRD2. Proc. Natl. Acad. Sci. USA.

[90] Shaw N., Zhao M., Cheng C., Xu H., Saarikettu J., Li Y., Da Y., Yao Z., Silvennoinen O., Yang J. (2007). The Multifunctional Human P100 Protein “hooks” Methylated Ligands. Nat. Struct. Mol. Biol..

[91] Jumper J., Evans R., Pritzel A., Green T., Figurnov M., Ronneberger O., Tunyasuvunakool K., Bates R., Žídek A., Potapenko A. (2021). Highly Accurate Protein Structure Prediction with AlphaFold. Nature.

[92] Varadi M., Anyango S., Deshpande M., Nair S., Natassia C., Yordanova G., Yuan D., Stroe O., Wood G., Laydon A. (2022). AlphaFold Protein Structure Database: Massively Expanding the Structural Coverage of Protein-Sequence Space with High-Accuracy Models. Nucleic Acids Res..

[93] Li C.-L., Yang W.-Z., Chen Y.-P., Yuan H.S. (2008). Structural and Functional Insights into Human Tudor-SN, a Key Component Linking RNA Interference and Editing. Nucleic Acids Res..

[94] Gutierrez-Beltran E., Denisenko T.V., Zhivotovsky B., Bozhkov P.V. (2016). Tudor Staphylococcal Nuclease: Biochemistry and Functions. Cell Death Differ..

[95] Ponting C.P. (1997). P100, a Transcriptional Coactivator, Is a Human Homologue of Staphylococcal Nuclease. Protein Sci..

[96] Caudy A.A., Ketting R.F., Hammond S.M., Denli A.M., Bathoorn A.M.P., Tops B.B.J., Silva J.M., Myers M.M., Hannon G.J., Plasterk R.H.A. (2003). A Micrococcal Nuclease Homologue in RNAi Effector Complexes. Nature.

[97] Hossain M.J., Korde R., Singh S., Mohmmed A., Dasaradhi P.V.N., Chauhan V.S., Malhotra P. (2008). Tudor Domain Proteins in Protozoan Parasites and Characterization of Plasmodium Falciparum Tudor Staphylococcal Nuclease. Int. J. Parasitol..

[98] Goulet I., Boisvenue S., Mokas S., Mazroui R., Cote J. (2008). TDRD3, a Novel Tudor Domain-Containing Protein, Localizes to Cytoplasmic Stress Granules. Hum. Mol. Genet..

[99] Chou H.-L., Tian L., Fukuda M., Kumamaru T., Okita T.W. (2019). The Role of RNA-Binding Protein OsTudor-SN in Post-Transcriptional Regulation of Seed Storage Proteins and Endosperm Development. Plant Cell Physiol..

[100] Wesche J., Kühn S., Kessler B.M., Salton M., Wolf A. (2017). Protein Arginine Methylation: A Prominent Modification and Its Demethylation. Cell. Mol. Life Sci..

[101] Métivier R., Penot G., Hübner M.R., Reid G., Brand H., Koš M., Gannon F. (2003). Estrogen Receptor-α Directs Ordered, Cyclical, and Combinatorial Recruitment of Cofactors on a Natural Target Promoter. Cell.

[102] Le Romancer M., Treilleux I., Leconte N., Robin-Lespinasse Y., Sentis S., Bouchekioua-Bouzaghou K., Goddard S., Gobert-Gosse S., Corbo L. (2008). Regulation of Estrogen Rapid Signaling through Arginine Methylation by PRMT1. Mol. Cell.

[103] Sakabe K., Hart G.W. (2010). O-GlcNAc Transferase Regulates Mitotic Chromatin Dynamics. J. Biol. Chem..

[104] Sylvestersen K.B., Horn H., Jungmichel S., Jensen L.J., Nielsen M.L. (2014). Proteomic Analysis of Arginine Methylation Sites in Human Cells Reveals Dynamic Regulation During Transcriptional Arrest. Mol. Cell. Proteom..

[105] Tsai W.-C., Gayatri S., Reineke L.C., Sbardella G., Bedford M.T., Lloyd R.E. (2016). Arginine Demethylation of G3BP1 Promotes Stress Granule Assembly. J. Biol. Chem..

[106] Wang Y., Wysocka J., Sayegh J., Lee Y.-H., Perlin J.R., Leonelli L., Sonbuchner L.S., McDonald C.H., Cook R.G., Dou Y. (2004). Human PAD4 Regulates Histone Arginine Methylation Levels via Demethylimination. Science.

[107] Wang S., Wang Y. (2013). Peptidylarginine Deiminases in Citrullination, Gene Regulation, Health and Pathogenesis. Biochim. Biophys. Acta (BBA)—Gene Regul. Mech..

[108] Hidaka Y., Hagiwara T., Yamada M. (2005). Methylation of the Guanidino Group of Arginine Residues Prevents Citrullination by Peptidylarginine Deiminase IV. FEBS Lett..

[109] Kearney P.L., Bhatia M., Jones N.G., Yuan L., Glascock M.C., Catchings K.L., Yamada M., Thompson P.R. (2005). Kinetic Characterization of Protein Arginine Deiminase 4: A Transcriptional Corepressor Implicated in the Onset and Progression of Rheumatoid Arthritis. Biochemistry.

[110] Raijmakers R., Zendman A.J.W., Egberts W.V., Vossenaar E.R., Raats J., Soede-Huijbregts C., Rutjes F.P.J.T., Van Veelen P.A., Drijfhout J.W., Pruijn G.J.M. (2007). Methylation of Arginine Residues Interferes with Citrullination by Peptidylarginine Deiminases in Vitro. J. Mol. Biol..

[111] Chang B., Chen Y., Zhao Y., Bruick R.K. (2007). JMJD6 Is a Histone Arginine Demethylase. Science.

[112] Liu W., Ma Q., Wong K., Li W., Ohgi K., Zhang J., Aggarwal A.K., Rosenfeld M.G. (2013). Brd4 and JMJD6-Associated Anti-Pause Enhancers in Regulation of Transcriptional Pause Release. Cell.

[113] Lawrence P., Conderino J.S., Rieder E. (2014). Redistribution of Demethylated RNA Helicase A during Foot-and-Mouth Disease Virus Infection: Role of Jumonji C-Domain Containing Protein 6 in RHA Demethylation. Virology.

[114] Tikhanovich I., Kuravi S., Artigues A., Villar M.T., Dorko K., Nawabi A., Roberts B., Weinman S.A. (2015). Dynamic Arginine Methylation of Tumor Necrosis Factor (TNF) Receptor-Associated Factor 6 Regulates Toll-like Receptor Signaling. J. Biol. Chem..

[115] Tsai W.-C., Reineke L.C., Jain A., Jung S.Y., Lloyd R.E. (2017). Histone Arginine Demethylase JMJD6 Is Linked to Stress Granule Assembly through Demethylation of the Stress Granule–Nucleating Protein G3BP1. J. Biol. Chem..

[116] Poulard C., Rambaud J., Hussein N., Corbo L., Le Romancer M. (2014). JMJD6 Regulates ERα Methylation on Arginine. PLoS ONE.

[117] Guo Z., Hu Y., Feng G., Valenzuela Ripoll C., Li Z., Cai S., Wang Q., Luo W., Li Q., Liang L. (2023). JMJD6 Protects against Isoproterenol-Induced Cardiac Hypertrophy via Inhibition of NF-κB Activation by Demethylating R149 of the P65 Subunit. Acta Pharmacol. Sin..

[118] Pesiridis G.S., Diamond E., Van Duyne G.D. (2009). Role of pICLn in Methylation of Sm Proteins by PRMT5. J. Biol. Chem..

[119] Arribas-Layton M., Dennis J., Bennett E.J., Damgaard C.K., Lykke-Andersen J. (2016). The C-Terminal RGG Domain of Human Lsm4 Promotes Processing Body Formation Stimulated by Arginine Dimethylation. Mol. Cell. Biol..

[120] Fürst J., Schedlbauer A., Gandini R., Garavaglia M.L., Saino S., Gschwentner M., Sarg B., Lindner H., Jakab M., Ritter M. (2005). ICln159 Folds into a Pleckstrin Homology Domain-like Structure. J. Biol. Chem..

[121] Paknia E., Chari A., Stark H., Fischer U. (2016). The Ribosome Cooperates with the Assembly Chaperone pICln to Initiate Formation of snRNPs. Cell Rep..

[122] Guderian G., Peter C., Wiesner J., Sickmann A., Schulze-Osthoff K., Fischer U., Grimmler M. (2011). RioK1, a New Interactor of Protein Arginine Methyltransferase 5 (PRMT5), Competes with pICln for Binding and Modulates PRMT5 Complex Composition and Substrate Specificity. J. Biol. Chem..

[123] Lacroix M., Messaoudi S.E., Rodier G., Le Cam A., Sardet C., Fabbrizio E. (2008). The Histone-binding Protein COPR5 Is Required for Nuclear Functions of the Protein Arginine Methyltransferase PRMT5. EMBO Rep..

[124] Mulvaney K.M., Blomquist C., Acharya N., Li R., Ranaghan M.J., O’Keefe M., Rodriguez D.J., Young M.J., Kesar D., Pal D. (2021). Molecular Basis for Substrate Recruitment to the PRMT5 Methylosome. Mol. Cell.

[125] Ho M.-C., Wilczek C., Bonanno J.B., Xing L., Seznec J., Matsui T., Carter L.G., Onikubo T., Kumar P.R., Chan M.K. (2013). Structure of the Arginine Methyltransferase PRMT5-MEP50 Reveals a Mechanism for Substrate Specificity. PLoS ONE.

[126] Liu F., Zhao X., Perna F., Wang L., Koppikar P., Abdel-Wahab O., Harr M.W., Levine R.L., Xu H., Tefferi A. (2011). JAK2V617F-Mediated Phosphorylation of PRMT5 Downregulates Its Methyltransferase Activity and Promotes Myeloproliferation. Cancer Cell.

[127] Nie M., Wang Y., Guo C., Li X., Wang Y., Deng Y., Yao B., Gui T., Ma C., Liu M. (2018). CARM1-Mediated Methylation of Protein Arginine Methyltransferase 5 Represses Human γ-Globin Gene Expression in Erythroleukemia Cells. J. Biol. Chem..

[128] Zhang H.-T., Zeng L.-F., He Q.-Y., Tao W.A., Zha Z.-G., Hu C.-D. (2016). The E3 Ubiquitin Ligase CHIP Mediates Ubiquitination and Proteasomal Degradation of PRMT5. Biochim. Biophys. Acta (BBA)—Mol. Cell Res..

[129] Singhroy D.N., Mesplède T., Sabbah A., Quashie P.K., Falgueyret J.-P., Wainberg M.A. (2013). Automethylation of Protein Arginine Methyltransferase 6 (PRMT6) Regulates Its Stability and Its Anti-HIV-1 Activity. Retrovirology.

